# Supplementary material for: Multi-omics analyses reveal that the gut microbiome and its metabolites promote milk fat synthesis in Zhongdian yak cows
Source: PeerJ. 2022 Dec 2;10:e14444. doi: 10.7717/peerj.14444 (PMC9744170; doi:10.7717/peerj.14444)
Supplement: Supplemental Information 13 [file peerj-10-14444-s013.zip › Web_Report/HTML/meta_lipidmaps_anno_categroy.xls.html]

meta\_lipidmaps\_anno\_categroy.xls


## meta\_lipidmaps\_anno\_categroy.xls

| CATEGORY | MAIN\_CLASS | Metabolites\_nums | Metabolites |
| --- | --- | --- | --- |
| Fatty Acyls [FA] | Docosanoids [FA04] | 127 | pos\_366 pos\_50 pos\_1252 pos\_82 pos\_563 pos\_513 pos\_1096 pos\_663 pos\_412 pos\_1005 pos\_2479 pos\_560 pos\_1982 pos\_653 pos\_884 pos\_288 pos\_2365 pos\_2744 pos\_709 pos\_1973 pos\_1570 pos\_775 pos\_2760 pos\_1173 pos\_577 pos\_2726 pos\_1490 pos\_1940 pos\_1659 pos\_157 pos\_1477 pos\_2666 pos\_2759 pos\_494 pos\_85 pos\_1253 pos\_809 pos\_779 pos\_114 pos\_1489 pos\_818 pos\_271 pos\_856 pos\_2337 pos\_459 pos\_1714 pos\_801 pos\_2026 pos\_200 pos\_240 pos\_2326 pos\_2741 pos\_450 pos\_1904 pos\_1311 pos\_604 pos\_2732 pos\_48 pos\_101 pos\_83 pos\_932 pos\_2166 pos\_1623 pos\_660 pos\_1803 pos\_392 pos\_961 pos\_554 pos\_2122 pos\_1314 pos\_87 pos\_362 pos\_805 pos\_723 pos\_2687 pos\_281 pos\_219 pos\_116 pos\_180 pos\_2107 pos\_1219 pos\_2628 pos\_478 pos\_607 pos\_177 pos\_273 pos\_830 pos\_2822 pos\_2626 pos\_1124 pos\_2573 pos\_265 pos\_148 pos\_2682 pos\_1590 pos\_689 pos\_1633 pos\_1554 pos\_2049 pos\_659 pos\_454 pos\_2372 pos\_1114 pos\_1150 pos\_2782 pos\_1030 pos\_186 pos\_541 pos\_1601 pos\_14 pos\_755 pos\_630 pos\_1190 pos\_695 pos\_2812 pos\_110 pos\_1705 pos\_499 pos\_1291 pos\_2842 pos\_1355 pos\_195 pos\_1577 pos\_373 pos\_1334 pos\_29 pos\_2696 |
| Fatty Acyls [FA] | Eicosanoids [FA03] | 64 | pos\_366 pos\_50 pos\_1253 pos\_659 pos\_809 pos\_454 pos\_723 pos\_1252 pos\_2372 pos\_82 pos\_271 pos\_2122 pos\_2782 pos\_2337 pos\_1096 pos\_1030 pos\_186 pos\_1714 pos\_1005 pos\_663 pos\_805 pos\_961 pos\_2687 pos\_14 pos\_541 pos\_2026 pos\_281 pos\_560 pos\_219 pos\_1601 pos\_1982 pos\_755 pos\_180 pos\_288 pos\_695 pos\_1219 pos\_2696 pos\_2812 pos\_478 pos\_607 pos\_110 pos\_177 pos\_450 pos\_1904 pos\_1705 pos\_709 pos\_499 pos\_1291 pos\_265 pos\_1973 pos\_2842 pos\_101 pos\_1355 pos\_2760 pos\_1173 pos\_83 pos\_2682 pos\_2726 pos\_577 pos\_2166 pos\_157 pos\_1590 pos\_1633 pos\_554 |
| Fatty Acyls [FA] | Fatty Acids and Conjugates [FA01] | 43 | pos\_366 pos\_659 pos\_809 pos\_454 pos\_1252 pos\_2372 pos\_82 pos\_271 pos\_1030 pos\_186 pos\_1714 pos\_1005 pos\_663 pos\_805 pos\_961 pos\_2687 pos\_14 pos\_2026 pos\_281 pos\_560 pos\_755 pos\_288 pos\_695 pos\_1219 pos\_2696 pos\_2812 pos\_478 pos\_450 pos\_1904 pos\_1705 pos\_709 pos\_499 pos\_1291 pos\_1973 pos\_1355 pos\_2760 pos\_1173 pos\_83 pos\_2726 pos\_157 pos\_1590 pos\_1633 pos\_554 |
| Fatty Acyls [FA] | Fatty acyl glycosides [FA13] | 105 | pos\_366 pos\_50 pos\_1252 pos\_82 pos\_563 pos\_513 pos\_1096 pos\_663 pos\_1005 pos\_2479 pos\_560 pos\_1982 pos\_884 pos\_288 pos\_2365 pos\_2744 pos\_709 pos\_1973 pos\_2760 pos\_1173 pos\_577 pos\_2726 pos\_1940 pos\_1659 pos\_157 pos\_2666 pos\_2759 pos\_494 pos\_1253 pos\_809 pos\_114 pos\_1489 pos\_271 pos\_856 pos\_2337 pos\_459 pos\_1714 pos\_801 pos\_2026 pos\_240 pos\_450 pos\_1904 pos\_604 pos\_2732 pos\_101 pos\_83 pos\_2166 pos\_1623 pos\_1803 pos\_392 pos\_961 pos\_554 pos\_2122 pos\_362 pos\_805 pos\_723 pos\_2687 pos\_281 pos\_219 pos\_116 pos\_180 pos\_2107 pos\_1219 pos\_2628 pos\_478 pos\_607 pos\_177 pos\_273 pos\_830 pos\_2822 pos\_2626 pos\_1124 pos\_2573 pos\_265 pos\_148 pos\_2682 pos\_1590 pos\_689 pos\_1633 pos\_1554 pos\_659 pos\_454 pos\_2372 pos\_1114 pos\_2782 pos\_1030 pos\_186 pos\_541 pos\_1601 pos\_14 pos\_755 pos\_630 pos\_695 pos\_2812 pos\_110 pos\_1705 pos\_499 pos\_1291 pos\_2842 pos\_1355 pos\_195 pos\_1577 pos\_373 pos\_29 pos\_2696 |
| Fatty Acyls [FA] | Fatty alcohols [FA05] | 124 | pos\_366 pos\_50 pos\_1252 pos\_82 pos\_563 pos\_513 pos\_1096 pos\_663 pos\_412 pos\_1005 pos\_2479 pos\_560 pos\_1982 pos\_653 pos\_884 pos\_288 pos\_2365 pos\_2744 pos\_709 pos\_1973 pos\_1570 pos\_775 pos\_2760 pos\_1173 pos\_577 pos\_2726 pos\_1490 pos\_1940 pos\_1659 pos\_157 pos\_2666 pos\_2759 pos\_494 pos\_85 pos\_1253 pos\_809 pos\_114 pos\_1489 pos\_818 pos\_271 pos\_856 pos\_2337 pos\_459 pos\_1714 pos\_801 pos\_2026 pos\_200 pos\_240 pos\_2741 pos\_450 pos\_1904 pos\_1311 pos\_604 pos\_2732 pos\_48 pos\_101 pos\_83 pos\_932 pos\_2166 pos\_1623 pos\_660 pos\_1803 pos\_392 pos\_961 pos\_554 pos\_2122 pos\_1314 pos\_87 pos\_362 pos\_805 pos\_723 pos\_2687 pos\_281 pos\_219 pos\_116 pos\_180 pos\_2107 pos\_1219 pos\_2628 pos\_478 pos\_607 pos\_177 pos\_273 pos\_830 pos\_2822 pos\_2626 pos\_1124 pos\_2573 pos\_265 pos\_148 pos\_2682 pos\_1590 pos\_689 pos\_1633 pos\_1554 pos\_2049 pos\_659 pos\_454 pos\_2372 pos\_1114 pos\_1150 pos\_2782 pos\_1030 pos\_186 pos\_541 pos\_1601 pos\_14 pos\_755 pos\_630 pos\_1190 pos\_695 pos\_2812 pos\_110 pos\_1705 pos\_499 pos\_1291 pos\_2842 pos\_1355 pos\_195 pos\_1577 pos\_373 pos\_1334 pos\_29 pos\_2696 |
| Fatty Acyls [FA] | Fatty aldehydes [FA06] | 50 | pos\_366 pos\_659 pos\_809 pos\_454 pos\_1252 pos\_2372 pos\_82 pos\_271 pos\_1030 pos\_186 pos\_1714 pos\_1005 pos\_663 pos\_805 pos\_961 pos\_2687 pos\_14 pos\_541 pos\_2026 pos\_281 pos\_560 pos\_219 pos\_755 pos\_180 pos\_288 pos\_695 pos\_1219 pos\_2696 pos\_2812 pos\_478 pos\_110 pos\_177 pos\_450 pos\_1904 pos\_1705 pos\_709 pos\_499 pos\_1291 pos\_265 pos\_1973 pos\_101 pos\_1355 pos\_2760 pos\_1173 pos\_83 pos\_2726 pos\_157 pos\_1590 pos\_1633 pos\_554 |
| Fatty Acyls [FA] | Fatty amides [FA08] | 80 | pos\_366 pos\_50 pos\_1252 pos\_82 pos\_513 pos\_1096 pos\_663 pos\_1005 pos\_560 pos\_1982 pos\_884 pos\_288 pos\_709 pos\_1973 pos\_2760 pos\_1173 pos\_577 pos\_2726 pos\_1940 pos\_157 pos\_1253 pos\_809 pos\_1489 pos\_271 pos\_856 pos\_2337 pos\_1714 pos\_2026 pos\_450 pos\_1904 pos\_101 pos\_83 pos\_2166 pos\_1623 pos\_1803 pos\_961 pos\_554 pos\_2122 pos\_362 pos\_805 pos\_723 pos\_2687 pos\_281 pos\_219 pos\_180 pos\_1219 pos\_478 pos\_607 pos\_177 pos\_273 pos\_2822 pos\_265 pos\_148 pos\_2682 pos\_1590 pos\_689 pos\_1633 pos\_659 pos\_454 pos\_2372 pos\_1114 pos\_2782 pos\_1030 pos\_186 pos\_541 pos\_1601 pos\_14 pos\_755 pos\_630 pos\_695 pos\_2812 pos\_110 pos\_1705 pos\_499 pos\_1291 pos\_2842 pos\_1355 pos\_373 pos\_29 pos\_2696 |
| Fatty Acyls [FA] | Fatty esters [FA07] | 104 | pos\_366 pos\_50 pos\_1252 pos\_82 pos\_563 pos\_513 pos\_1096 pos\_663 pos\_1005 pos\_2479 pos\_560 pos\_1982 pos\_884 pos\_288 pos\_2365 pos\_2744 pos\_709 pos\_1973 pos\_2760 pos\_1173 pos\_577 pos\_2726 pos\_1940 pos\_1659 pos\_157 pos\_2666 pos\_2759 pos\_494 pos\_1253 pos\_809 pos\_1489 pos\_271 pos\_856 pos\_2337 pos\_459 pos\_1714 pos\_801 pos\_2026 pos\_240 pos\_450 pos\_1904 pos\_604 pos\_2732 pos\_101 pos\_83 pos\_2166 pos\_1623 pos\_1803 pos\_392 pos\_961 pos\_554 pos\_2122 pos\_362 pos\_805 pos\_723 pos\_2687 pos\_281 pos\_219 pos\_116 pos\_180 pos\_2107 pos\_1219 pos\_2628 pos\_478 pos\_607 pos\_177 pos\_273 pos\_830 pos\_2822 pos\_2626 pos\_1124 pos\_2573 pos\_265 pos\_148 pos\_2682 pos\_1590 pos\_689 pos\_1633 pos\_1554 pos\_659 pos\_454 pos\_2372 pos\_1114 pos\_2782 pos\_1030 pos\_186 pos\_541 pos\_1601 pos\_14 pos\_755 pos\_630 pos\_695 pos\_2812 pos\_110 pos\_1705 pos\_499 pos\_1291 pos\_2842 pos\_1355 pos\_195 pos\_1577 pos\_373 pos\_29 pos\_2696 |
| Fatty Acyls [FA] | Hydrocarbons [FA11] | 134 | pos\_366 pos\_50 pos\_1252 pos\_82 pos\_563 pos\_513 pos\_1096 pos\_663 pos\_412 pos\_1005 pos\_2479 pos\_560 pos\_1982 pos\_653 pos\_884 pos\_288 pos\_2365 pos\_46 pos\_2744 pos\_709 pos\_25 pos\_1973 pos\_1570 pos\_775 pos\_2760 pos\_1173 pos\_577 pos\_2726 pos\_1490 pos\_1940 pos\_1659 pos\_157 pos\_1477 pos\_463 pos\_2666 pos\_2759 pos\_494 pos\_85 pos\_1253 pos\_809 pos\_779 pos\_114 pos\_1489 pos\_818 pos\_271 pos\_856 pos\_2337 pos\_459 pos\_1714 pos\_801 pos\_2026 pos\_200 pos\_240 pos\_2326 pos\_2741 pos\_450 pos\_1904 pos\_1311 pos\_604 pos\_2732 pos\_48 pos\_101 pos\_83 pos\_932 pos\_2166 pos\_1623 pos\_660 pos\_1803 pos\_392 pos\_961 pos\_554 pos\_2122 pos\_726 pos\_1314 pos\_87 pos\_362 pos\_805 pos\_723 pos\_2687 pos\_581 pos\_281 pos\_219 pos\_116 pos\_180 pos\_2107 pos\_1219 pos\_2628 pos\_478 pos\_607 pos\_177 pos\_273 pos\_830 pos\_2822 pos\_2626 pos\_1124 pos\_2573 pos\_265 pos\_148 pos\_2682 pos\_1590 pos\_689 pos\_1633 pos\_1554 pos\_2049 pos\_659 pos\_454 pos\_2372 pos\_1114 pos\_1150 pos\_2782 pos\_72 pos\_1030 pos\_186 pos\_541 pos\_1601 pos\_14 pos\_755 pos\_630 pos\_1190 pos\_695 pos\_2812 pos\_110 pos\_1705 pos\_499 pos\_1291 pos\_2842 pos\_1355 pos\_195 pos\_1577 pos\_373 pos\_1334 pos\_29 pos\_2696 pos\_436 |
| Fatty Acyls [FA] | Octadecanoids [FA02] | 131 | pos\_366 pos\_50 pos\_1252 pos\_82 pos\_563 pos\_513 pos\_1096 pos\_663 pos\_412 pos\_1005 pos\_2479 pos\_560 pos\_1982 pos\_653 pos\_884 pos\_288 pos\_2365 pos\_2744 pos\_709 pos\_25 pos\_1973 pos\_1570 pos\_775 pos\_2760 pos\_1173 pos\_577 pos\_2726 pos\_1490 pos\_1940 pos\_1659 pos\_157 pos\_1477 pos\_463 pos\_2666 pos\_2759 pos\_494 pos\_85 pos\_1253 pos\_809 pos\_779 pos\_114 pos\_1489 pos\_818 pos\_271 pos\_856 pos\_2337 pos\_459 pos\_1714 pos\_801 pos\_2026 pos\_200 pos\_240 pos\_2326 pos\_2741 pos\_450 pos\_1904 pos\_1311 pos\_604 pos\_2732 pos\_48 pos\_101 pos\_83 pos\_932 pos\_2166 pos\_1623 pos\_660 pos\_1803 pos\_392 pos\_961 pos\_554 pos\_2122 pos\_726 pos\_1314 pos\_87 pos\_362 pos\_805 pos\_723 pos\_2687 pos\_281 pos\_219 pos\_116 pos\_180 pos\_2107 pos\_1219 pos\_2628 pos\_478 pos\_607 pos\_177 pos\_273 pos\_830 pos\_2822 pos\_2626 pos\_1124 pos\_2573 pos\_265 pos\_148 pos\_2682 pos\_1590 pos\_689 pos\_1633 pos\_1554 pos\_2049 pos\_659 pos\_454 pos\_2372 pos\_1114 pos\_1150 pos\_2782 pos\_1030 pos\_186 pos\_541 pos\_1601 pos\_14 pos\_755 pos\_630 pos\_1190 pos\_695 pos\_2812 pos\_110 pos\_1705 pos\_499 pos\_1291 pos\_2842 pos\_1355 pos\_195 pos\_1577 pos\_373 pos\_1334 pos\_29 pos\_2696 pos\_436 |
| Fatty Acyls [FA] | Oxygenated hydrocarbons [FA12] | 128 | pos\_366 pos\_50 pos\_1252 pos\_82 pos\_563 pos\_513 pos\_1096 pos\_663 pos\_412 pos\_1005 pos\_2479 pos\_560 pos\_1982 pos\_653 pos\_884 pos\_288 pos\_2365 pos\_2744 pos\_709 pos\_25 pos\_1973 pos\_1570 pos\_775 pos\_2760 pos\_1173 pos\_577 pos\_2726 pos\_1490 pos\_1940 pos\_1659 pos\_157 pos\_1477 pos\_2666 pos\_2759 pos\_494 pos\_85 pos\_1253 pos\_809 pos\_779 pos\_114 pos\_1489 pos\_818 pos\_271 pos\_856 pos\_2337 pos\_459 pos\_1714 pos\_801 pos\_2026 pos\_200 pos\_240 pos\_2326 pos\_2741 pos\_450 pos\_1904 pos\_1311 pos\_604 pos\_2732 pos\_48 pos\_101 pos\_83 pos\_932 pos\_2166 pos\_1623 pos\_660 pos\_1803 pos\_392 pos\_961 pos\_554 pos\_2122 pos\_1314 pos\_87 pos\_362 pos\_805 pos\_723 pos\_2687 pos\_281 pos\_219 pos\_116 pos\_180 pos\_2107 pos\_1219 pos\_2628 pos\_478 pos\_607 pos\_177 pos\_273 pos\_830 pos\_2822 pos\_2626 pos\_1124 pos\_2573 pos\_265 pos\_148 pos\_2682 pos\_1590 pos\_689 pos\_1633 pos\_1554 pos\_2049 pos\_659 pos\_454 pos\_2372 pos\_1114 pos\_1150 pos\_2782 pos\_1030 pos\_186 pos\_541 pos\_1601 pos\_14 pos\_755 pos\_630 pos\_1190 pos\_695 pos\_2812 pos\_110 pos\_1705 pos\_499 pos\_1291 pos\_2842 pos\_1355 pos\_195 pos\_1577 pos\_373 pos\_1334 pos\_29 pos\_2696 |
| Glycerolipids [GL] | Diradylglycerols [GL02] | 2 | pos\_1442 pos\_250 |
| Glycerolipids [GL] | Glycosylmonoradylglycerols [GL04] | 3 | pos\_1442 pos\_250 pos\_527 |
| Glycerolipids [GL] | Triradylglycerols [GL03] | 4 | pos\_1442 pos\_914 pos\_250 pos\_527 |
| Glycerophospholipids [GP] | CDP-Glycerols [GP13] | 67 | pos\_1339 pos\_1207 pos\_2801 pos\_115 pos\_1593 pos\_1017 pos\_1876 pos\_423 pos\_2341 pos\_1684 pos\_2159 pos\_718 pos\_2195 pos\_2151 pos\_1863 pos\_1143 pos\_1301 pos\_889 pos\_2239 pos\_1503 pos\_2138 pos\_2769 pos\_1603 pos\_331 pos\_2755 pos\_1963 pos\_2521 pos\_1898 pos\_1257 pos\_1168 pos\_2035 pos\_2305 pos\_1161 pos\_1270 pos\_2615 pos\_705 pos\_2743 pos\_1865 pos\_2809 pos\_1430 pos\_1926 pos\_441 pos\_1295 pos\_2752 pos\_2175 pos\_111 pos\_2559 pos\_1125 pos\_1819 pos\_825 pos\_2632 pos\_2779 pos\_2641 pos\_1007 pos\_763 pos\_2119 pos\_2787 pos\_2683 pos\_1604 pos\_1423 pos\_260 pos\_338 pos\_1067 pos\_549 pos\_1664 pos\_1795 pos\_2639 |
| Glycerophospholipids [GP] | Glycerophosphates [GP10] | 40 | pos\_1339 pos\_2801 pos\_115 pos\_1017 pos\_423 pos\_2341 pos\_2159 pos\_2151 pos\_1863 pos\_1143 pos\_1301 pos\_1503 pos\_2138 pos\_1603 pos\_331 pos\_1963 pos\_1898 pos\_1257 pos\_1168 pos\_2035 pos\_2305 pos\_1161 pos\_705 pos\_2809 pos\_441 pos\_1295 pos\_2752 pos\_111 pos\_2559 pos\_1125 pos\_1819 pos\_825 pos\_2641 pos\_1007 pos\_2119 pos\_2787 pos\_2683 pos\_1604 pos\_260 pos\_1067 |
| Glycerophospholipids [GP] | Glycerophosphocholines [GP01] | 51 | pos\_1339 pos\_1207 pos\_2801 pos\_115 pos\_1017 pos\_423 pos\_2341 pos\_1684 pos\_2159 pos\_718 pos\_2151 pos\_1863 pos\_1143 pos\_1301 pos\_1503 pos\_2138 pos\_2769 pos\_1603 pos\_331 pos\_2755 pos\_1963 pos\_1898 pos\_1257 pos\_1168 pos\_2035 pos\_2305 pos\_1161 pos\_1270 pos\_2615 pos\_705 pos\_1865 pos\_2809 pos\_1430 pos\_441 pos\_1295 pos\_2752 pos\_111 pos\_2559 pos\_1125 pos\_1819 pos\_825 pos\_2641 pos\_1007 pos\_763 pos\_2119 pos\_2787 pos\_2683 pos\_1604 pos\_260 pos\_1067 pos\_1664 |
| Glycerophospholipids [GP] | Glycerophosphoethanolamines [GP02] | 19 | pos\_1339 pos\_1017 pos\_423 pos\_2341 pos\_2159 pos\_2151 pos\_1143 pos\_1503 pos\_331 pos\_1963 pos\_1257 pos\_2035 pos\_2305 pos\_1295 pos\_111 pos\_2559 pos\_2641 pos\_1007 pos\_1067 |
| Glycerophospholipids [GP] | Glycerophosphoglycerols [GP04] | 62 | pos\_1339 pos\_1207 pos\_2801 pos\_115 pos\_1593 pos\_1017 pos\_423 pos\_2341 pos\_1684 pos\_2159 pos\_718 pos\_2195 pos\_2151 pos\_1863 pos\_1143 pos\_1301 pos\_889 pos\_1503 pos\_2138 pos\_2769 pos\_1603 pos\_331 pos\_2755 pos\_1963 pos\_1898 pos\_1257 pos\_1168 pos\_2035 pos\_2305 pos\_1161 pos\_1270 pos\_2615 pos\_705 pos\_2743 pos\_1865 pos\_2809 pos\_1430 pos\_1926 pos\_441 pos\_1295 pos\_2752 pos\_111 pos\_2559 pos\_1125 pos\_1819 pos\_825 pos\_2632 pos\_2641 pos\_1007 pos\_763 pos\_2119 pos\_2787 pos\_2683 pos\_1604 pos\_1423 pos\_260 pos\_338 pos\_1067 pos\_549 pos\_1664 pos\_1795 pos\_2639 |
| Glycerophospholipids [GP] | Glycerophosphoglycerophosphates [GP05] | 63 | pos\_1339 pos\_1207 pos\_2801 pos\_115 pos\_1593 pos\_1017 pos\_1876 pos\_423 pos\_2341 pos\_1684 pos\_2159 pos\_718 pos\_2195 pos\_2151 pos\_1863 pos\_1143 pos\_1301 pos\_889 pos\_1503 pos\_2138 pos\_2769 pos\_1603 pos\_331 pos\_2755 pos\_1963 pos\_1898 pos\_1257 pos\_1168 pos\_2035 pos\_2305 pos\_1161 pos\_1270 pos\_2615 pos\_705 pos\_2743 pos\_1865 pos\_2809 pos\_1430 pos\_1926 pos\_441 pos\_1295 pos\_2752 pos\_111 pos\_2559 pos\_1125 pos\_1819 pos\_825 pos\_2632 pos\_2641 pos\_1007 pos\_763 pos\_2119 pos\_2787 pos\_2683 pos\_1604 pos\_1423 pos\_260 pos\_338 pos\_1067 pos\_549 pos\_1664 pos\_1795 pos\_2639 |
| Glycerophospholipids [GP] | Glycerophosphoinositols [GP06] | 56 | pos\_1339 pos\_1207 pos\_2801 pos\_115 pos\_1017 pos\_423 pos\_2341 pos\_1684 pos\_2159 pos\_718 pos\_2151 pos\_1863 pos\_1143 pos\_1301 pos\_889 pos\_1503 pos\_2138 pos\_2769 pos\_1603 pos\_331 pos\_2755 pos\_1963 pos\_1898 pos\_1257 pos\_1168 pos\_2035 pos\_2305 pos\_1161 pos\_1270 pos\_2615 pos\_705 pos\_1865 pos\_2809 pos\_1430 pos\_441 pos\_1295 pos\_2752 pos\_111 pos\_2559 pos\_1125 pos\_1819 pos\_825 pos\_2632 pos\_2641 pos\_1007 pos\_763 pos\_2119 pos\_2787 pos\_2683 pos\_1604 pos\_1423 pos\_260 pos\_1067 pos\_1664 pos\_1795 pos\_2639 |
| Glycerophospholipids [GP] | Glycerophosphoserines [GP03] | 31 | pos\_1339 pos\_2801 pos\_1017 pos\_423 pos\_2341 pos\_2159 pos\_2151 pos\_1143 pos\_1301 pos\_1503 pos\_2138 pos\_331 pos\_1963 pos\_1898 pos\_1257 pos\_2035 pos\_2305 pos\_1161 pos\_2809 pos\_1295 pos\_111 pos\_2559 pos\_1125 pos\_1819 pos\_2641 pos\_1007 pos\_2119 pos\_2787 pos\_2683 pos\_1604 pos\_1067 |
| Polyketides [PK] | Aromatic polyketides [PK13] | 61 | pos\_2194 pos\_802 pos\_1193 pos\_203 pos\_1289 pos\_80 pos\_685 pos\_2120 pos\_179 pos\_612 pos\_783 pos\_225 pos\_486 pos\_1312 pos\_2301 pos\_257 pos\_605 pos\_2596 pos\_56 pos\_1859 pos\_27 pos\_1101 pos\_1763 pos\_130 pos\_278 pos\_1033 pos\_100 pos\_1930 pos\_142 pos\_2080 pos\_890 pos\_1104 pos\_187 pos\_1557 pos\_348 pos\_427 pos\_89 pos\_1038 pos\_376 pos\_2361 pos\_2281 pos\_1406 pos\_315 pos\_297 pos\_33 pos\_335 pos\_381 pos\_2385 pos\_90 pos\_2830 pos\_567 pos\_117 pos\_1499 pos\_248 pos\_1607 pos\_389 pos\_107 pos\_232 pos\_382 pos\_2043 pos\_112 |
| Polyketides [PK] | Flavonoids [PK12] | 57 | pos\_2194 pos\_802 pos\_1193 pos\_203 pos\_1289 pos\_80 pos\_685 pos\_2120 pos\_179 pos\_612 pos\_783 pos\_225 pos\_486 pos\_1312 pos\_2301 pos\_257 pos\_605 pos\_2596 pos\_56 pos\_1859 pos\_27 pos\_1101 pos\_1763 pos\_278 pos\_1033 pos\_100 pos\_1930 pos\_142 pos\_2080 pos\_890 pos\_187 pos\_1557 pos\_348 pos\_427 pos\_89 pos\_376 pos\_2361 pos\_2281 pos\_1406 pos\_315 pos\_297 pos\_33 pos\_335 pos\_381 pos\_2385 pos\_90 pos\_567 pos\_117 pos\_1499 pos\_248 pos\_1607 pos\_389 pos\_107 pos\_232 pos\_382 pos\_2043 pos\_112 |
| Polyketides [PK] | Linear tetracyclines [PK07] | 60 | pos\_2194 pos\_802 pos\_1193 pos\_203 pos\_1289 pos\_80 pos\_685 pos\_2120 pos\_179 pos\_612 pos\_783 pos\_225 pos\_486 pos\_1312 pos\_2301 pos\_257 pos\_605 pos\_2596 pos\_56 pos\_1859 pos\_27 pos\_1101 pos\_1763 pos\_278 pos\_1033 pos\_100 pos\_1930 pos\_142 pos\_2080 pos\_890 pos\_1104 pos\_187 pos\_1557 pos\_348 pos\_427 pos\_89 pos\_1038 pos\_376 pos\_2361 pos\_2281 pos\_1406 pos\_315 pos\_297 pos\_33 pos\_335 pos\_381 pos\_2385 pos\_90 pos\_2830 pos\_567 pos\_117 pos\_1499 pos\_248 pos\_1607 pos\_389 pos\_107 pos\_232 pos\_382 pos\_2043 pos\_112 |
| Polyketides [PK] | Macrolides and lactone polyketides [PK04] | 59 | pos\_2194 pos\_802 pos\_1193 pos\_203 pos\_1289 pos\_80 pos\_685 pos\_2120 pos\_179 pos\_612 pos\_783 pos\_225 pos\_486 pos\_1312 pos\_2301 pos\_257 pos\_605 pos\_2596 pos\_56 pos\_1859 pos\_27 pos\_1101 pos\_1763 pos\_278 pos\_1033 pos\_100 pos\_1930 pos\_142 pos\_2080 pos\_890 pos\_187 pos\_1557 pos\_348 pos\_427 pos\_89 pos\_1038 pos\_376 pos\_2361 pos\_2281 pos\_1406 pos\_315 pos\_297 pos\_33 pos\_335 pos\_381 pos\_2385 pos\_90 pos\_2830 pos\_567 pos\_117 pos\_1499 pos\_248 pos\_1607 pos\_389 pos\_107 pos\_232 pos\_382 pos\_2043 pos\_112 |
| Polyketides [PK] | Non-ribosomal peptide/polyketide hybrids [PK14] | 66 | pos\_2194 pos\_802 pos\_1193 pos\_203 pos\_22 pos\_1289 pos\_80 pos\_685 pos\_2120 pos\_179 pos\_612 pos\_783 pos\_225 pos\_486 pos\_1312 pos\_2301 pos\_257 pos\_605 pos\_2596 pos\_56 pos\_1859 pos\_27 pos\_2826 pos\_1101 pos\_1763 pos\_130 pos\_278 pos\_1033 pos\_643 pos\_100 pos\_1930 pos\_142 pos\_2080 pos\_890 pos\_1104 pos\_187 pos\_1557 pos\_348 pos\_427 pos\_89 pos\_1038 pos\_376 pos\_2361 pos\_2811 pos\_2281 pos\_1406 pos\_315 pos\_297 pos\_33 pos\_335 pos\_381 pos\_2385 pos\_90 pos\_2830 pos\_567 pos\_117 pos\_1499 pos\_248 pos\_1607 pos\_389 pos\_107 pos\_232 pos\_667 pos\_382 pos\_2043 pos\_112 |
| Polyketides [PK] | Phenolic lipids [PK15] | 65 | pos\_2194 pos\_802 pos\_1193 pos\_203 pos\_22 pos\_1289 pos\_80 pos\_685 pos\_2120 pos\_179 pos\_612 pos\_783 pos\_225 pos\_486 pos\_1312 pos\_2301 pos\_257 pos\_605 pos\_2596 pos\_56 pos\_1859 pos\_27 pos\_2826 pos\_1101 pos\_1763 pos\_130 pos\_278 pos\_1033 pos\_643 pos\_100 pos\_1930 pos\_142 pos\_2080 pos\_890 pos\_1104 pos\_187 pos\_1557 pos\_348 pos\_427 pos\_89 pos\_1038 pos\_376 pos\_2361 pos\_2281 pos\_1406 pos\_315 pos\_297 pos\_33 pos\_335 pos\_381 pos\_2385 pos\_90 pos\_2830 pos\_567 pos\_117 pos\_1499 pos\_248 pos\_1607 pos\_389 pos\_107 pos\_232 pos\_667 pos\_382 pos\_2043 pos\_112 |
| Polyketides [PK] | Polyenes [PK06] | 67 | pos\_2194 pos\_802 pos\_892 pos\_1193 pos\_203 pos\_22 pos\_1289 pos\_80 pos\_685 pos\_2120 pos\_179 pos\_612 pos\_783 pos\_225 pos\_486 pos\_1312 pos\_2301 pos\_257 pos\_605 pos\_2596 pos\_56 pos\_1859 pos\_27 pos\_2826 pos\_1101 pos\_1763 pos\_130 pos\_278 pos\_1033 pos\_643 pos\_100 pos\_1930 pos\_142 pos\_2080 pos\_890 pos\_1104 pos\_187 pos\_1557 pos\_348 pos\_427 pos\_89 pos\_1038 pos\_376 pos\_2361 pos\_2811 pos\_2281 pos\_1406 pos\_315 pos\_297 pos\_33 pos\_335 pos\_381 pos\_2385 pos\_90 pos\_2830 pos\_567 pos\_117 pos\_1499 pos\_248 pos\_1607 pos\_389 pos\_107 pos\_232 pos\_667 pos\_382 pos\_2043 pos\_112 |
| Prenol Lipids [PR] | Isoprenoids [PR01] | 36 | pos\_147 pos\_735 pos\_1787 pos\_106 pos\_2321 pos\_76 pos\_1783 pos\_97 pos\_769 pos\_699 pos\_301 pos\_129 pos\_2832 pos\_467 pos\_2823 pos\_178 pos\_2029 pos\_199 pos\_2729 pos\_1077 pos\_1799 pos\_55 pos\_283 pos\_1579 pos\_9 pos\_2308 pos\_1265 pos\_151 pos\_166 pos\_34 pos\_78 pos\_44 pos\_153 pos\_86 pos\_49 pos\_191 |
| Prenol Lipids [PR] | Polyprenols [PR03] | 37 | pos\_147 pos\_735 pos\_1787 pos\_106 pos\_2321 pos\_76 pos\_1783 pos\_97 pos\_769 pos\_699 pos\_301 pos\_129 pos\_2832 pos\_467 pos\_2823 pos\_178 pos\_2029 pos\_199 pos\_2729 pos\_1077 pos\_1799 pos\_55 pos\_283 pos\_1579 pos\_1282 pos\_9 pos\_2308 pos\_1265 pos\_151 pos\_166 pos\_34 pos\_78 pos\_44 pos\_153 pos\_86 pos\_49 pos\_191 |
| Prenol Lipids [PR] | Quinones and hydroquinones [PR02] | 42 | pos\_147 pos\_735 pos\_1752 pos\_1787 pos\_106 pos\_2321 pos\_76 pos\_1783 pos\_97 pos\_769 pos\_2466 pos\_699 pos\_301 pos\_129 pos\_2832 pos\_467 pos\_2823 pos\_178 pos\_2029 pos\_199 pos\_2729 pos\_1077 pos\_1799 pos\_55 pos\_2853 pos\_283 pos\_1579 pos\_1282 pos\_9 pos\_1844 pos\_2308 pos\_1265 pos\_1383 pos\_151 pos\_166 pos\_34 pos\_78 pos\_44 pos\_153 pos\_86 pos\_49 pos\_191 |
| Sphingolipids [SP] | Ceramides [SP02] | 4 | pos\_28 pos\_1079 pos\_1983 pos\_2030 |
| Sphingolipids [SP] | Neutral glycosphingolipids [SP05] | 5 | pos\_1248 pos\_1079 pos\_28 pos\_2030 pos\_1983 |
| Sphingolipids [SP] | Phosphosphingolipids [SP03] | 11 | pos\_1741 pos\_1248 pos\_1256 pos\_2538 pos\_1079 pos\_28 pos\_2818 pos\_2851 pos\_2030 pos\_813 pos\_1983 |
| Sphingolipids [SP] | Sphingoid bases [SP01] | 9 | pos\_1248 pos\_1256 pos\_1079 pos\_28 pos\_2818 pos\_2851 pos\_2030 pos\_813 pos\_1983 |
| Sterol Lipids [ST] | Bile acids and derivatives [ST04] | 6 | pos\_10 pos\_1956 pos\_3 pos\_1055 pos\_396 pos\_1234 |
| Sterol Lipids [ST] | Secosteroids [ST03] | 63 | pos\_1480 pos\_10 pos\_1148 pos\_1003 pos\_2481 pos\_1900 pos\_562 pos\_631 pos\_1109 pos\_2761 pos\_8 pos\_871 pos\_3 pos\_1055 pos\_396 pos\_1169 pos\_1050 pos\_606 pos\_1407 pos\_1234 pos\_919 pos\_2135 pos\_2735 pos\_2155 pos\_359 pos\_1617 pos\_2072 pos\_1218 pos\_270 pos\_438 pos\_2020 pos\_198 pos\_687 pos\_1204 pos\_2353 pos\_416 pos\_1956 pos\_1146 pos\_669 pos\_2054 pos\_1191 pos\_1637 pos\_2074 pos\_507 pos\_517 pos\_57 pos\_1868 pos\_1638 pos\_788 pos\_1447 pos\_1155 pos\_350 pos\_1158 pos\_2298 pos\_2542 pos\_2063 pos\_360 pos\_1467 pos\_1170 pos\_2392 pos\_594 pos\_618 pos\_2180 |
| Sterol Lipids [ST] | Steroid conjugates [ST05] | 62 | pos\_1480 pos\_10 pos\_1148 pos\_1003 pos\_2481 pos\_1900 pos\_562 pos\_631 pos\_1109 pos\_2761 pos\_8 pos\_871 pos\_3 pos\_1055 pos\_396 pos\_1169 pos\_1050 pos\_606 pos\_1407 pos\_1234 pos\_919 pos\_2135 pos\_2735 pos\_2155 pos\_359 pos\_1617 pos\_2072 pos\_1218 pos\_270 pos\_438 pos\_2020 pos\_198 pos\_687 pos\_1204 pos\_2353 pos\_416 pos\_1956 pos\_1146 pos\_669 pos\_2054 pos\_1191 pos\_1637 pos\_2074 pos\_507 pos\_517 pos\_57 pos\_1868 pos\_1638 pos\_788 pos\_1447 pos\_1155 pos\_1158 pos\_2298 pos\_2542 pos\_2063 pos\_360 pos\_1467 pos\_1170 pos\_2392 pos\_594 pos\_618 pos\_2180 |
| Sterol Lipids [ST] | Steroids [ST02] | 31 | pos\_1480 pos\_10 pos\_1148 pos\_1003 pos\_2481 pos\_1900 pos\_562 pos\_3 pos\_1055 pos\_396 pos\_1169 pos\_1407 pos\_1234 pos\_1617 pos\_2072 pos\_270 pos\_438 pos\_2020 pos\_1204 pos\_2353 pos\_1956 pos\_1146 pos\_507 pos\_517 pos\_1868 pos\_1638 pos\_2298 pos\_2542 pos\_2063 pos\_594 pos\_618 |
| Sterol Lipids [ST] | Sterols [ST01] | 51 | pos\_1480 pos\_10 pos\_1148 pos\_1003 pos\_2481 pos\_1900 pos\_562 pos\_1109 pos\_2761 pos\_3 pos\_1055 pos\_396 pos\_1169 pos\_1050 pos\_606 pos\_1407 pos\_1234 pos\_2735 pos\_359 pos\_1617 pos\_2072 pos\_1218 pos\_270 pos\_438 pos\_2020 pos\_198 pos\_687 pos\_1204 pos\_2353 pos\_416 pos\_1956 pos\_1146 pos\_2054 pos\_1191 pos\_1637 pos\_507 pos\_517 pos\_57 pos\_1868 pos\_1638 pos\_788 pos\_1155 pos\_1158 pos\_2298 pos\_2542 pos\_2063 pos\_360 pos\_1170 pos\_2392 pos\_594 pos\_618 |
